# Dynamic intratubular biomineralization following root canal obturation with pozzolan‐based mineral trioxide aggregate sealer cement

**DOI:** 10.1002/sca.21240

**Published:** 2015-07-14

**Authors:** Yeon‐Jee Yoo, Seung‐Ho Baek, Kee‐Yeon Kum, Won‐Jun Shon, Kyung‐Mi Woo, WooCheol Lee

**Affiliations:** ^1^ Department of Conservative Dentistry, School of Dentistry, Dental Research InstituteSeoul National UniversitySeoulKorea; ^2^ Department of Pharmacology and Dental Therapeutics, School of Dentistry, Dental Research InstituteSeoul National UniversitySeoulKorea

**Keywords:** biomineralization, dentinal tubule, pozzolan‐based MTA sealer, pre‐crystallization seeds, scanning electron microscopy

## Abstract

The application of mineral trioxide aggregates (MTA) cement during the root canal obturation is gaining concern due to its bioactive characteristic to form an apatite in dentinal tubules. In this regard, this study was to assess the biomineralization of dentinal tubules following root canal obturation by using pozzolan‐based (Pz‐) MTA sealer cement (EndoSeal MTA, Maruchi). Sixty curved roots (mesiobuccal, distobuccal) from human maxillary molars were instrumented and prepared for root canal obturation. The canals were obturated with gutta‐percha (GP) and Pz‐MTA sealer by using continuous wave of condensation technique. Canals obturated solely with ProRoot MTA (Dentsply Tulsa Dental) or Pz‐MTA sealer were used for comparison. In order to evaluate the biomineralization ability under different conditions, the PBS pretreatment before the root canal obturation was performed in each additional samples. At dentin‐material interfaces, the extension of intratubular biomineralization was analyzed using scanning electron microscopy (SEM) and energy dispersive spectroscopy. When the root canal was obturated with GP and Pz‐MTA sealer, enhanced biomineralization of the dentinal tubules beyond the penetrated sealer tag was confirmed under the SEM observation (p < 0.05). Mineralized apatite structures (calcium/phosphorous ratio, 1.45–1.89) connecting its way through the dentinal tubules were detected at 350–400 μm from the tubule orifice, and the pre‐crystallization seeds were also observed along the intra‐ and/or inter‐tubular collagen fiber. Intratubular biomineralization depth was significantly enhanced in all PBS pretreated canals (p < 0.05). Pz‐MTA cement can be used as a promising bioactive root canal sealer to enhance biomineralization of dentinal tubules under controlled environment. SCANNING 38:50–56, 2016. © 2015 The Authors. *Scanning* Published by Wiley Periodicals, Inc.

## Introduction

Endodontic treatment is an ongoing process to eliminate infection source and to create a fluid‐tight seal of the root canal system (Siqueira, 2001). Theoretically a root canal filling material which can completely seal the root canal system would be of ideal in practice. However, several studies confirmed that currently available root canal obturation materials such as gutta‐percha (GP) and/or polymer‐based materials showed incomplete sealing even with the aid of sealers or dentin bonding systems (Zmener *et al*., 2008; Santos *et al*., 2010; Brosco *et al*., 2010; Punia *et al*., 2011).

Mineral trioxide aggregate (MTA) has been widely used in variety of applications including root‐end filling, perforation repair, or apical/coronal sealing material during regenerative endodontic procedures (Parirokh and Torabinejad, 2010). The most favorable property, leaving its biologic properties aside, is the superior sealing ability which comes from the water‐resistant final product after hydration. This sealing ability of MTA is largely attributable to its bioactive capacity to form an apatite layer when it is in contact with phosphate‐containing physiological fluids (Tay *et al*., 2007; Reyes‐Carmona *et al*., 2009; Gandolfi *et al*., 2010; Han *et al*., 2010). Such characteristic features of MTA appear to be important in biomineralization of dentinal tubules for enhanced sealing of the root canal system (Tay *et al*., 2007; Reyes‐Carmona *et al*., 2010; Yoo *et al*., 2014), thus makes it a good candidate for root canal filling material of choice.

However, MTA cannot be recommended as a routine orthograde root canal filling material because the sandy property and irretrievability of the substance (Bogen and Kuttler, 2009) have made it challenging to be used in a complicated root canal system. Inadequate water‐to‐powder ratio, insufficient packing also impedes adaptation of MTA to the canal wall (Fridland and Rosado, 2003; El‐Ma'aita *et al*., 2012; Saghiri *et al*., 2012). In order to overcome such limitations of MTA as a root canal filling material, recent study has utilized MTA sealer cement during the root canal filling procedure (Camilleri *et al*., 2011).

EndoSeal MTA (Maruchi, Wonju, Korea), a finely pulverized pozzolan‐based MTA was recently introduced. The pozzolan cement, the main component of this sealer, gets cementitious properties after pozzolanic reaction which includes calcium hydroxide and water, and enables sufficient flow of the pre‐mixed substrate though injection tip with adequate working consistency. The favorable mechanical characteristics such as fast setting time (around 4 min), higher washout resistance than other commercially available MTAs, and biologic effects including biocompatibility, mineralization potential, and odontogenic effect of the pozzolan cement had been previously reported (Choi *et al*., 2013; Jang *et al*., 2013a,2013b; Park *et al*., 2014; Song *et al*., 2014). However, no study has confirmed the intratubular biomineralization ability of this sealer material when it is applied in the root canal yet. Therefore, this study was aimed to investigate and compare the biomineralization ability of this pozzolan‐based (Pz‐) MTA sealer cement under various root canal obturation conditions.

## Materials and Methods

### Tooth Preparation

**Figure 1:**
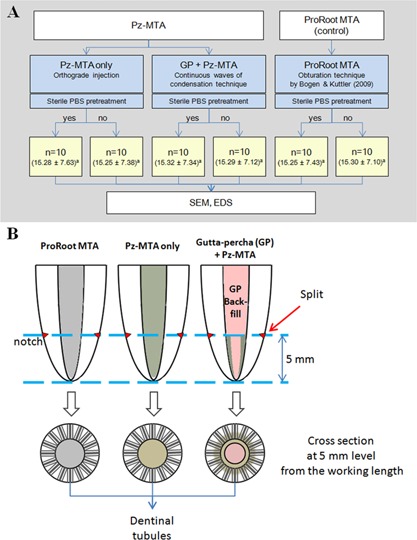
A: Schematic diagram of the experimental groups. The number in parenthesis shows the mean and standard deviation of the canal curvature of each set of roots, and their same lowercase superscripts show no significant differences among the mean values of the canal curvatures (p > 0.05). B: Schematic diagram of the root specimen preparation for SEM evaluation. MTA, mineral trioxide aggregate; Pz‐MTA, pozzolan‐based mineral trioxide aggregate; GP, gutta‐percha; PBS, phosphate buffered saline; SEM, scanning electron microscopy; EDS, energy dispersive spectroscopy.

The protocol of this study was approved by the Institutional Review Board of the Seoul National University Dental Hospital, Seoul, Korea. A total sample size of 56 roots was calculated to be sufficient to detect significant differences (alpha at level 0.05, 90% power). Sixty curved roots (mesiobuccal and distobuccal roots) less than 30 degree (Schneider, 1971) with fully formed apices from human maxillary molars were used in this study. Teeth with root cracks or defects confirmed under a microscopic evaluation (OPMI Pico; Carl Zeiss, Germany) were excluded from the study. All teeth were radiographically examined to evaluate the canal curvature, by measuring the angle between the long axis of the root and the line connecting the point that begins to move away from the long axis of the root to the apex (Schneider, 1971). The overall mean canal curvature was 15.28 ± 7.08 degree, and the roots were assigned to 6 sets (10 canals each) according to the canal curvature by block randomization (Fig. 1(A)).

The teeth were accessed with #4 round carbide burs and Endo‐Z burs. The canal patency was gained using a size #10 stainless‐steel (SS) K‐file (Dentsply Maillefer, Ballaigues, Switzerland) until the tips were visible at the apical foramen. The working length was determined as 1 mm short from the measured length. After coronal flaring using Gates Glidden burs #2 to #4 (Komet, Rock Hill, SC), root canals were instrumented to an apical size of #35 and 0.06 taper with a crown‐down technique using ProFile 0.04 and 0.06 Ni‐Ti rotary instruments (Dentsply Maillefer). The root canals were irrigated with 2 ml of 5.25% sodium hypochlorite (NaOCl) solution between instrumentation, and immersed with 17% ethylenediaminetetraacetic acid (EDTA) solution (pH 7.2) for 1 min before final flush with 2.5 ml of 5.25% NaOCl solution. All irrigants were activated by using ultrasonic devices (P5 Newtron® XS; Satelec, Acteon group, Mėrignac, France). Then, the canals were copiously rinsed with sterile distilled water and dried with sterile paper points.

For the root canal obturation procedure, 10 canals were obturated with MAF size‐ and taper‐ tailored GP cone and Pz‐MTA sealer (EndoSeal MTA) by using continuous wave of condensation technique (CW). For the comparison control, another 10 canals were obturated solely with Pz‐MTA per se. The pre‐mixed Pz‐MTA sealer cement was released via injection syringe and tip system, and the final coronal portion was tidied up with SS hand pluggers. The canals (n = 10) filled with ProRoot MTA (Dentsply Tulsa Dental, Tulsa, OK) according to the obturation technique suggested by Bogen and Kuttler (2009) were also used as a positive control. The ProRoot MTA was mixed with distilled water according to the manufacturer's instructions and placed incrementally with a carrier gun. An SS K‐file, 1 or 2 sizes smaller than master apical file (MAF) was used to compact the apical 3–4 mm, then a progression of K‐files sizing upward incrementally were used for further compaction. The final coronal portion was tamped by using SS hand pluggers to complete the root canal obturation.

For the phosphate buffered saline (PBS) pretreatment, additional 10 canals were assigned to each experimental group. They were immersed in sterile PBS solution for a minute and dried with sterile paper points before root canal filling.

Following the obturation, the teeth were stored at 37°C with 100% humidity for a day to allow complete setting of the filling materials, and sealed the access cavities using intermediate restorative material (IRM; Dentsply Caulk, Milford, DE). The teeth were stored at 37°C with 100% humidity until further analysis.

### Assessment of Dentinal Tubule Biomineralization

After 12 weeks, the specimens were evaluated by scanning electron microscopy (SEM; S‐4700, Hitachi, Tokyo, Japan) to characterize microstructural variations of the dentinal tubules. Each tooth was embedded in an acrylic block, and each mesiobuccal or distobuccal root was separated from the teeth with a slow‐speed, water‐cooled diamond saw (Isomet Low Speed Saw; Buehler, Lake Bluff, IL). The separated roots were split in horizontal direction for cross‐section analysis at 5 mm level from the apex (Fig. 1(B)). Root segments were briefly washed in distilled water and sputter coated with platinum for SEM observation at an accelerating voltage of 15 kV. At the interface of main canal and obturation material, the depths of material penetration into dentinal tubules and intratubular mineralization were recorded. The elemental composition of intratubular mineralized precipitates were analyzed by using energy dispersive spectroscopy (EDS; 7200‐H, Horiba, Northampton, England).

### Statistical Analyses

The data were analyzed with one‐way ANOVA and Tuckey *post hoc* test with SPSS software (SPSS Inc., Chicago, IL) to assess the differences among experimental groups. For each group, the effect of PBS pretreatment on the intratubular biomineralization was investigated using a two‐sample t‐test. The significance level was set at *α* = 0.05.

## Results

**Figure 2:**
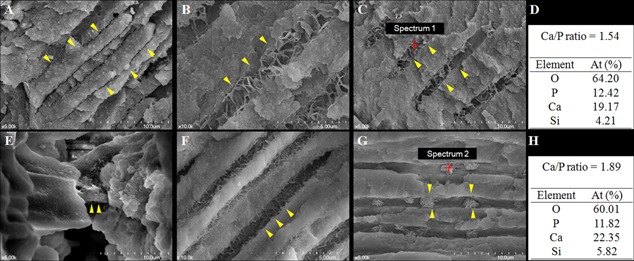
Representative scanning electron microscope images of gutta‐percha with pozzolan‐based (Pz‐) MTA sealer cement‐ (A–D) or ProRoot MTA‐ (E–H) filled roots with phosphate buffered saline pretreatment. A: The Pz‐MTA sealer cement penetrated into the dentinal tubules (arrowheads) at orifice level (×5,000). B: Further biomineralized dentinal tubules (arrowheads) beyond penetrated Pz‐MTA cement at 50–100 µm distance showing densely packed dentinal tubules with organized apatite nanoprecipitates (×10,000). C: Successively biomineralized dentinal tubules (arrowheads) at 100–150 µm distance (×5,000). D: The semiquantitative chemical composition showing Ca/P ratio of the pointed area (red) of (C). E: Intermediate layer of ProRoot MTA connecting the material and dentinal tubule (arrowheads) at orifice level (×5,000). F: Biomineralized dentinal tubules at 50–100 µm distance (×10,000). G: Biomineralized dentinal tubules at 100–150 µm distance (×5,000). The agglomerated precipitates induced by ProRoot MTA (arrowheads) sparsely clogged the dentinal tubules. H: The semiquantitative chemical composition showing Ca/P ratio of the pointed area (red) of (G).

The scanning analysis of GP with Pz‐MTA sealer obturated samples showed the direct tubular penetration of Pz‐MTA, and further formation of apatite crystals densely packing the dentinal tubules were detected (Fig. 2(A–C)). On the other hand, a close adaptation of the material to the main canals and intratubular biomineralization near the tubule orifices were observed in the ProRoot MTA obturation samples (Fig. 2(E–G)). The mineralized structure connecting the material‐dentin interface was confirmed at the orifice level, although there was a lacking of direct penetration of ProRoot MTA into the tubules. The patterns of apatite crystallization appeared similar at the entrance of dentinal tubules in both GP with Pz‐MTA sealer and ProRoot MTA obturated samples (Fig. 2(B and F)). However, they simultaneously changed along the tubule pathway; the agglomerated precipitates sparsely clogged the dentinal tubules in ProRoot MTA obturated samples (Fig. 2(G)), whereas continuous and successive crystallization along the tubule pathway was observed in GP with Pz‐MTA sealer group (Fig. 2(C)). EDS evaluation indicated that the intratubular mineralized precipitates from all groups contained primarily calcium (Ca), phosphorous (P), oxygen, and trailed amount of silica with similar Ca/P ratio (1.45–1.89) (Fig. 2(D and H)).

**Figure 3:**
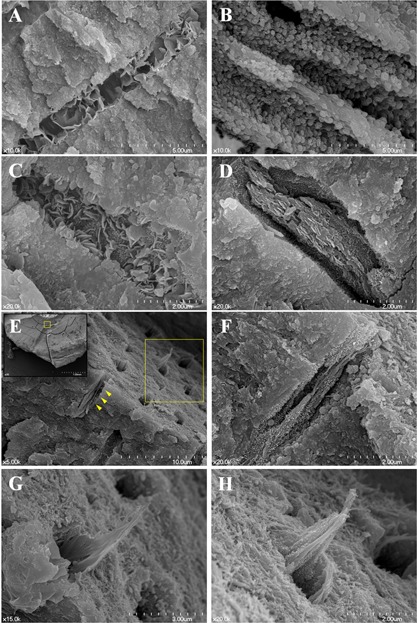
A–D: Diverse nanocrystallographs of pozzolan‐based (Pz‐) MTA sealer cement induced intratubular biomineralization. A: Organized nanoflakes (×10,000), (B) microsphere (×10,000), (C) mixed (×20,000), or (D) organized plates (×20000) are intergrown and exploited to seal the dentinal tubules. (E‐F) Successive intratubular biomineralization of gutta‐percha and Pz‐MTA sealer filled canals. E: Boxed area (yellow) of left upper lower magnification image (×35) from the horizontal split specimen obturated with gutta‐percha and Pz‐MTA sealer cement showing the interface at 350–400 μm distance from dentinal tubule orifice (×5,000). (F) The pre‐crystallization seeds observed along the collagen fiber (arrowheads of (E), ×20,000). (G, H) A higher magnification of upper right boxed area of (E) showing successive intratubular biomineralization in either (G) plate‐like form or (H) agglomeration of precipitation seeds along the collagen fibrils (×20,000). Scanning electron microscope images.

In GP with Pz‐MTA sealer group, there was a variety of precipitate nanostructures, mainly petals and flakes, in stratified or organized spherical form, or mixed (Fig. 3(A–D)). Interestingly, mineralized apatite structures connecting its way through the dentinal tubules were confirmed at 350–400 μm from the tubule orifice, and the pre‐crystallization seeds were also observed along the intra‐ and/or inter‐tubular collagen fiber (Fig. 3(E–H)).

The depth of intratubular mineralization is presented in Table I. The roots in GP with Pz‐MTA sealer group demonstrated the direct tubular penetration of the sealer with significantly greater depth of dentinal tubule biomineralization than the other groups (p < 0.05). Pretreatment with PBS significantly promoted the biomineralization depth in all groups (p < 0.05). The roots solely obturated with either of ProRoot MTA or Pz‐MTA sealer showed the minimum biomineralization depth without penetration of the materials into the tubules.

**Table I:** Depths of material penetration into the dentinal tubules and intratubular mineralization (mean ± standard deviation)

Root canal obturation material	GP with Pz‐MTA sealer	Pz‐MTA sealer only	ProRoot MTA only
Tubular penetration depth (μm)	23.77 ± 2.48	Not detected	Not detected
Intratubular mineralization depth (μm)			
PBS pretreatment			
No	350.25 ± 36.50^Ab^	62.55 ± 9.56^Bb^	68.20 ± 11.20^Bb^
Yes	392.69 ± 39.43^Aa^	98.12 ± 14.45^Ba^	130.51 ± 20.21^Ba^

GP, gutta percha; MTA, mineral trioxide aggregate; PBS, phosphate buffered saline; Pz‐MTA, pozzolan‐derived mineral trioxide aggregate. Same uppercase alphabet superscripts in row show no significant differences among the mean values of experimental groups (p > 0.05). Same lowercase alphabet superscripts in column show no significant effect of PBS pretreatment on the mean values of intratubular mineralization within each group (p > 0.05).

## Discussion

Seeking for the better root canal filling material is the utmost concern of the clinicians. However the technological advancement of the obturation method had not been shown to have a statistically relevant impact on treatment outcome (Peng *et al*., 2007). The clinical radiographs with densely filled root canals do not support the biologically sealed root canal system from surrounding periapical tissue. In fact, several methodologies such as micro‐computed tomography proposed to assess the sealing ability of root canal filling materials did not fully provide sufficient amount of information about the sealing of the root canal system. In this regard, confirmation of the dentinal tubule mineralization may provide the secondhand evidence showing the possible ultimate sealing of root canal system, and for this purpose, the present study was conducted to extrapolate and compare the biomineralization capacity of currently available Pz‐MTA cement with enhanced clinical conveniences.

In the present study, the Pz‐MTA sealer cement showed further intratubular biomineralization up to significantly deeper level of the tubules. This provides compelling evidence of Pz‐MTA as a bioactive root canal sealer when it was coupled with core material (GP) and vertical condensation pressure. In addition, PBS pretreatment before final obturation enhanced intratubular mineralization of both Pz‐MTA sealer‐ (110–150%) and ProRoot MTA‐ (130–190%) obturated canals. Although the intratubular mineralization depth of MTA was rather limited when compared to previous researches (Reyes‐Carmona *et al*., 2009; Reyes‐Carmona *et al*., 2010) in which the specimens were immersed continuously in regularly refreshed PBS solution, the strategic importance to boost biomineralization ability is the incorporation of phosphate ion as an initial precipitation seed. Preconditioning with the phosphate ions derived from PBS soaking sequence might have enhanced the nucleation formation, which is known as polymer‐induced liquid precursor (PILP) process (Gower, 2008). Phosphate anions in PBS are considered to make the intratubular environment even more labile in PILP process, and enhance the formation of the prenucleation cluster and its subsequent crystal growth. The collagen fibers exposed after smear layer removal with EDTA pretreatment also might have been directed the crystallization process once it is infiltrated with the amorphous precursors. However, there have not been any reports on the clinical use and tubular biomineralization inducing ability of PBS as a final soaking solution before root canal obturation. In this regard, the results of our SEM analysis (Fig. 3) is the first report of biomineralized apatite confirmed at 350‐400 μm level of dentinal tubules with variety of precipitate nanostructures, such as petals and flakes, in stratified or organized spherical form.

It is noteworthy that although bulk obturated materials were closely adapted to the canal wall, occasionally clogging the tubule orifices as previously reported (Bird *et al*., 2012), they could not penetrate into the tubules regardless of their different particle sizes. Rather, they showed the mineralized tag‐like structures connecting the material‐dentin interface at the orifice level. These structures are supposed to be the flocculated crystals formed on the material surface, which have been grown from the precursor–precipitation phase (Reyes‐Carmona *et al*., 2009; Reyes‐Carmona *et al*., 2010). In fact, precedent researches reported that such tag‐like structures were the result of biomineralization potential of Portland cement and MTA, which could be enhanced by the interaction with phosphate‐containing solution (Reyes‐Carmona *et al*., 2009; Reyes‐Carmona *et al*., 2010). However, the tags found in dentinal tubules of GP with Pz‐MTA sealer group are the results of material penetration aided by the vertical condensation pressure transmitted via thermoplasticized gutta‐percha, and are clearly distinct from such “tag‐like structures” in the other groups.

Further, the small particle size would have contributed to induce more stable precursors for guiding an effective diffusion of the ions than the higher molecular weight particles of ProRoot MTA (Huang *et al*., 2008). The mean particle size for white ProRoot MTA is 10 µm, with all particles being smaller than 50 µm (Komabayashi and Spangberg, 2008). The slurry made from such aggregates of particles become rheopectic when orthograde‐filled in root canals. In contrast, finely pulverized Pz‐MTA cement, with a mean particle size of 1.5 µm, becomes thixotropic when the material is released via needle tip and further compressed by vertical pressure. It then infiltrates or grouts toward dentinal tubules to form sealer tags and apatite precursors for further intratubular biomineralization. Such stable precursors may induce the propagation of crystallization along the dentinal tubules by secondary nucleation among individual nanoparticles of the disordered phase, providing successive biomineralization densified into deeper tubules. In fact, ProRoot MTA treated teeth showed sparsely clogging discrete agglomerates in limited depth.

The crystallographs of Pz‐MTA cement‐induced precipitates were also notable. They were in various shapes, relatively smaller in size than those induced by ProRoot MTA, within elemental composition of Ca/P ratio similar to hydroxyapatite. Among the various apatite structures, the particles with a grain size less than 100 nm in at least one direction have higher surface activity and ultrafine structure, resulting in enhanced bioactivity than coarser crystals (Vallet‐Regi and Gonzalez‐Calbet, 2004). We could confirm the seamless flow of mineralization precipitates in GP with Pz‐MTA sealer group, extended beyond 300 μm‐depth regardless of the PBS pretreatment. In that, Pz‐MTA cement as a sealer presented a favorable biomineralization pattern for the sealing of root canal system, while the canals solely filled with Pz‐MTA sealer or ProRoot MTA lacked such fine structures. The in‐depth investigation on the correlation among crystallography and elemental composition of Pz‐MTA cement‐induced biomineralization of dentinal tubules requires further researches.

Within the limitations of this study, the use of Pz‐MTA cement as a sealer in conjunction with well‐fit gutta‐percha cone and vertical pressure resulted in consistent dentinal tubule biomineralization. Preconditioning with PBS before root canal obturation promoted PILP process, and led to enhanced biomineralization of the dentinal tubules beyond the penetrated Pz‐MTA sealer tag in various crystallographs. The Pz‐MTA cement as a root canal sealer thus figuratively renders a new possibility of bio‐tight sealing of the root canal system.
